# Cutaneous viral infection risk with oral Janus kinase inhibitors in atopic dermatitis: An exploratory single-center retrospective cohort study

**DOI:** 10.1016/j.jdin.2026.09.008

**Published:** 2026-10-01

**Authors:** Emi Sato, Naoko Obonai, Mayuko Iwata, Shiori Katayama, Hiroki Shimizu, Kotaro Ito, Shinichi Imafuku

**Affiliations:** a Department of Dermatology, Faculty of Medicine, Fukuoka University, Fukuoka, Japan; b Ito Dermatology Clinic, Kitsuki, Oita, Japan

**Keywords:** abrocitinib, atopic dermatitis, baricitinib, cutaneous viral infection, Janus kinase inhibitor, real-world data, upadacitinib

*To the Editor:* Oral Janus kinase inhibitors (JAKi) are effective systemic treatments for moderate-to-severe atopic dermatitis (AD), but infection remains an important safety concern.^1^, ^2^, ^3^, ^4^ We conducted an exploratory single-center retrospective cohort study to evaluate whether cutaneous viral infection risk differs among oral JAKi used for AD.

We reviewed 152 patients with AD treated with abrocitinib (ABR) (*n* = 57), baricitinib (BARI) (*n* = 54), or upadacitinib (UPA) (*n* = 41) between 2021 and 2026. The primary outcome was time to first cutaneous viral infection after treatment initiation, including herpes simplex virus, varicella-zoster virus, human papillomavirus, and molluscum contagiosum virus infections. Events were based on dermatologist-documented clinical diagnoses supported by medical record descriptions, prescriptions, or procedure records; no patients received daily antiviral prophylaxis. Because baseline characteristics differed across groups, particularly for high-dose treatment, JAKi-naïve status, prior cutaneous viral infection history, and treatment duration (Table I; Supplementary Table I, available via Mendeley at https://data.mendeley.com/datasets/g676yf7ygm/1), we performed Kaplan-Meier analysis, multivariable Cox regression, individual-drug sensitivity analysis, and propensity score‑matched analyses. Additional ascertainment, vaccination, propensity score, balance, and proportional hazards assessments are provided in the Supplementary Data, available via Mendeley at https://data.mendeley.com/datasets/g676yf7ygm/1.

**Table I:** Patient characteristics by treatment group

Characteristic	ABR (*n* = 57)	BARI (*n* = 54)	UPA (*n* = 41)	*P* value
Age, y	39.0 [24.0-51.0]	41.5 [24.0-53.0]	42.0 [25.0-52.0]	.952
Male sex, *n* (%)	30 (52.6)	35 (64.8)	27 (65.9)	.303
AD duration, y	20.0 [10.0-36.0]	17.5 [7.0-37.0]	26.0 [16.0-45.0]	.065
Baseline EASI	21.0 [17.0-32.0]	18.0 [16.0-23.0]	24.0 [16.0-35.0]	.101
High-dose treatment, *n* (%)	15 (26.3)	0 (0.0)	16 (39.0)	<.001
Combination of systemic therapies, *n* (%)	2 (3.5)	2 (3.7)	4 (9.8)	.425
JAKi-naïve, *n* (%)	43 (75.4)	48 (88.9)	26 (63.4)	.012
Prior history of cutaneous viral infection, *n* (%)	11 (19.3)	8 (14.8)	16 (39.0)	.018
Treatment duration, d	133.0 [68.0-251.0]	243.5 [105.5-432.8]	295.0 [180.0-493.0]	<.001

Continuous variables are shown as median [IQR], and categorical variables as *n* (%). *P* values were calculated using the χ^2^ test or Fisher’s exact test for categorical variables and the Kruskal-Wallis test for continuous variables.

*ABR*, Abrocitinib; *AD*, atopic dermatitis; *BARI*, baricitinib; *EASI*, Eczema Area and Severity Index; *JAKi*, Janus kinase inhibitors; *UPA*, upadacitinib.

The crude incidence rate of cutaneous viral infection was lowest in the ABR group (20.47 per 100 person-years), followed by the BARI (34.26 per 100 person-years) and UPA (45.27 per 100 person-years) groups (Supplementary Fig 1 and Table II, available via Mendeley at https://data.mendeley.com/datasets/g676yf7ygm/1). Subtype-specific analyses are provided in the Supplementary Data, available via Mendeley at https://data.mendeley.com/datasets/g676yf7ygm/1 (Supplementary Figs 2 and 3, available via Mendeley at https://data.mendeley.com/datasets/g676yf7ygm/1). Kaplan-Meier analysis showed significant differences in cutaneous viral infection-free survival among the 3 groups (log-rank *P* = .0128; Fig 1). In the primary multivariable Cox model (Supplementary Table III, available via Mendeley at https://data.mendeley.com/datasets/g676yf7ygm/1), BARI/UPA treatment was associated with a higher hazard of cutaneous viral infection than ABR (hazard ratio [HR], 3.08; 95% confidence interval [CI], 1.23-7.73; *P* = .016), and prior cutaneous viral infection history was also independently associated with the outcome (HR, 5.94; 95% CI, 3.01-11.70; *P* < .001). In an individual-drug model, both BARI (HR, 3.46; 95% CI, 1.20-10.01; *P* = .022) and UPA (HR, 2.84; 95% CI, 1.05-7.72; *P* = .040) showed higher hazards than ABR. Propensity score distributions showed acceptable but incomplete overlap (Supplementary Fig IV, available via Mendeley at https://data.mendeley.com/datasets/g676yf7ygm/1), and all post-match absolute standardized mean differences were <0.10 (Supplementary Table IV and Fig 5, available via Mendeley at https://data.mendeley.com/datasets/g676yf7ygm/1). The association remained significant in the full-matched (HR, 3.90; 95% CI, 1.14-13.27; *P* = .030) and optimal-matched cohorts (HR, 2.63; 95% CI, 1.06-6.53; *P* = .037) (Supplementary Table V, available via Mendeley at https://data.mendeley.com/datasets/g676yf7ygm/1). Schoenfeld residual-based tests showed no evidence of violation of the proportional hazards assumption (Supplementary Table VI, available via Mendeley at https://data.mendeley.com/datasets/g676yf7ygm/1).

**Fig 1:**
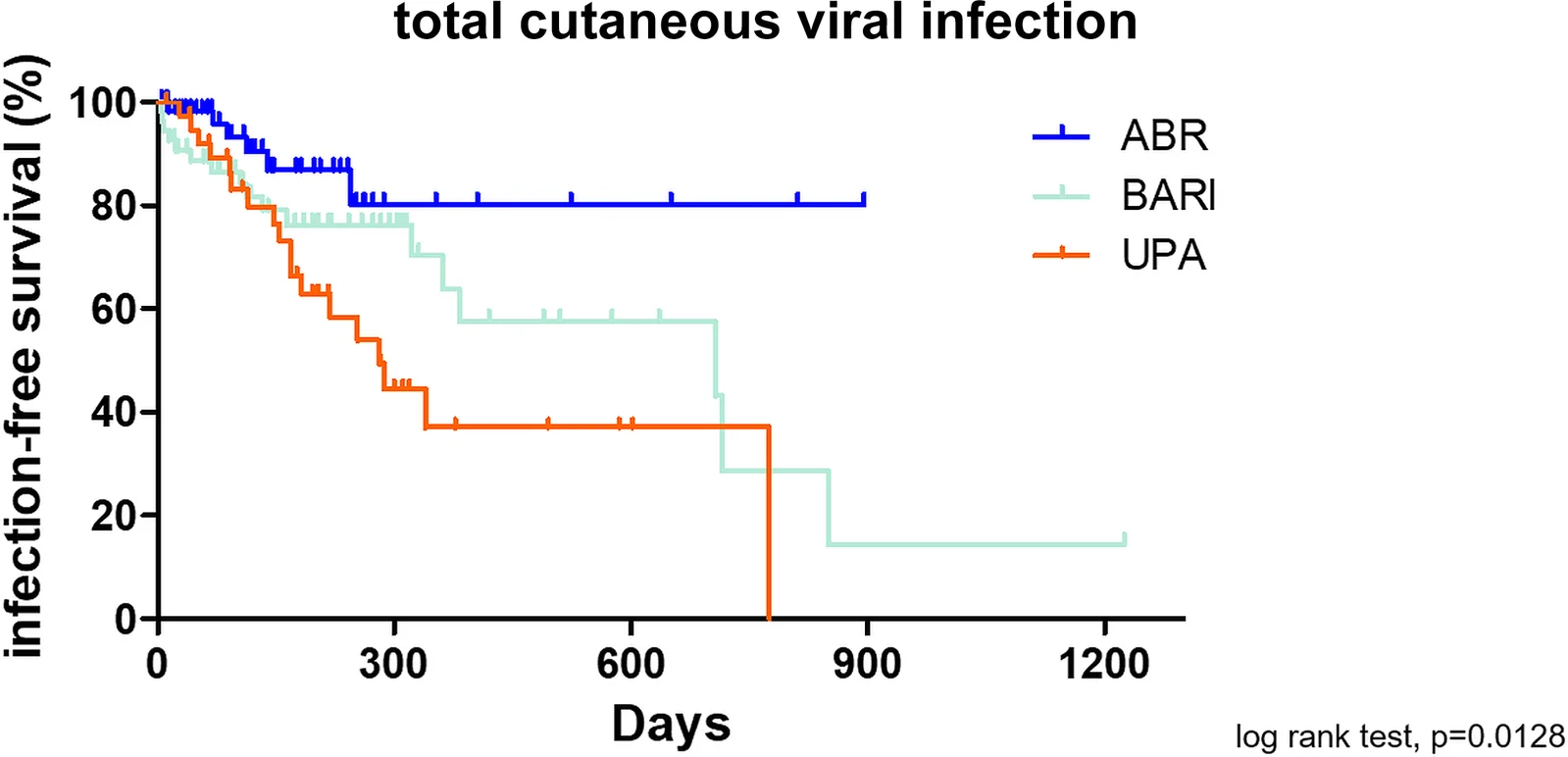
Kaplan-Meier curves for cutaneous viral infection-free survival according to oral Janus kinase inhibitor in patients with atopic dermatitis. Kaplan-Meier analysis was used to estimate the cumulative probability of remaining free from cutaneous viral infection in patients treated with abrocitinib (ABR), baricitinib (BARI), or upadacitinib (UPA). Differences among the 3 groups were evaluated using the log-rank test. *Tick marks* indicate censored observations.

These findings suggest an exploratory association between oral JAKi type and cutaneous viral infection risk in this cohort. They should not be interpreted as evidence of treatment-selection superiority or a mechanism related to Janus kinase selectivity. Limitations include the retrospective single-center design, nonrandom prescribing, calendar-era and dose differences, prior JAKi exposure, differential monitoring, residual confounding, and limited subtype-specific event numbers. Larger multicenter studies are needed to confirm these findings.

### Declaration of generative AI and AI-assisted technologies in the writing process

Artificial intelligence was used only for language editing support. The authors reviewed and approved the final manuscript and take full responsibility for its content.

## Conflicts of interest

Dr Sato has received personal fees, including fees for lectures, chairing, and/or advisory board participation, from AbbVie, Eli Lilly, Pfizer, Sanofi, LEO Pharma, Maruho, Torii, Sun Pharma, UCB, Taiho, Tanabe, Boehringer Ingelheim, and Novartis. Dr Shimizu has received personal fees, including fees for lectures and/or chairing, from UCB, AbbVie, Eli Lilly, Sanofi, Maruho, and Johnson & Johnson. Dr Ito has received personal fees, including fees for lectures, chairing, and/or advisory board participation, from AbbVie, Eli Lilly, Pfizer, Sanofi, LEO Pharma, Maruho, Sato, Bristol Myers Squibb, Torii, Sun Pharma, UCB, and Johnson & Johnson. Dr Imafuku has received personal fees, including fees for lectures, chairing, and/or advisory board participation, from AbbVie, Eli Lilly, Pfizer, Sanofi, LEO Pharma, Maruho, Sato, Bristol Myers Squibb, Torii, Sun Pharma, UCB, Johnson & Johnson, Amgen, Kyowa Kirin, GlaxoSmithKline, Boehringer Ingelheim, Novartis, Otsuka, and Taiho. Naoko Obonai, Mayuko Iwata, and Dr Katayama have no conflicts of interest to declare.
